# Advancing application of satellite remote sensing technologies for linking atmospheric and built environment to health

**DOI:** 10.3389/fpubh.2023.1270033

**Published:** 2023-11-15

**Authors:** Yuxuan Tian, Mengshan Duan, Xiangfen Cui, Qun Zhao, Senlin Tian, Yichao Lin, Weicen Wang

**Affiliations:** ^1^Faculty of Environmental Science and Engineering, Kunming University of Science and Technology, Kunming, Yunnan, China; ^2^Guizhou Research Institute of Coal Mine Design Co., Ltd., Guiyang, China; ^3^China Academy of Urban Planning Design, Beijing, China

**Keywords:** satellite remote sensing monitoring, atmospheric exposure science, land-cover monitoring, built environment, public health, geographical health

## Abstract

**Background:**

The intricate interplay between human well-being and the surrounding environment underscores contemporary discourse. Within this paradigm, comprehensive environmental monitoring holds the key to unraveling the intricate connections linking population health to environmental exposures. The advent of satellite remote sensing monitoring (SRSM) has revolutionized traditional monitoring constraints, particularly limited spatial coverage and resolution. This innovation finds profound utility in quantifying land covers and air pollution data, casting new light on epidemiological and geographical investigations. This dynamic application reveals the intricate web connecting public health, environmental pollution, and the built environment.

**Objective:**

This comprehensive review navigates the evolving trajectory of SRSM technology, casting light on its role in addressing environmental and geographic health issues. The discussion hones in on how SRSM has recently magnified our understanding of the relationship between air pollutant exposure and population health. Additionally, this discourse delves into public health challenges stemming from shifts in urban morphology.

**Methods:**

Utilizing the strategic keywords “SRSM,” “air pollutant health risk,” and “built environment,” an exhaustive search unfolded across prestigious databases including the China National Knowledge Network (CNKI), PubMed and Web of Science. The Citespace tool further unveiled interconnections among resultant articles and research trends.

**Results:**

Synthesizing insights from a myriad of articles spanning 1988 to 2023, our findings unveil how SRMS bridges gaps in ground-based monitoring through continuous spatial observations, empowering global air quality surveillance. High-resolution SRSM advances data precision, capturing multiple built environment impact factors. Its application to epidemiological health exposure holds promise as a pioneering tool for contemporary health research.

**Conclusion:**

This review underscores SRSM’s pivotal role in enriching geographic health studies, particularly in atmospheric pollution domains. The study illuminates how SRSM overcomes spatial resolution and data loss hurdles, enriching environmental monitoring tools and datasets. The path forward envisions the integration of cutting-edge remote sensing technologies, novel explorations of urban-public health associations, and an enriched assessment of built environment characteristics on public well-being.

## Introduction

Exposure to atmospheric pollutants has emerged as a significant risk factor contributing to the global burden of disease. The rapid economic growth and urbanization witnessed over the past decades have led to substantial emissions of particulate matter (PM) into the atmosphere, primarily from the combustion of coal and fossil fuels (1, 2). Of particular concern is PM with an aerodynamic diameter of less than 2.5 microns (i.e., PM_2.5_), which has been identified as a hazardous component in modern societies. Additionally, gaseous pollutants such as NO_x_, SO_2_, O_3_, and CO have also been concerned for their adverse influence on human health, causing significant and irreversible harm to human well-being (3–5). Surprisingly, despite efforts to mitigate global environmental and occupational exposure risks between 1990 and 2019, the exposure risks associated with atmospheric PM and O_3_ showed an alarming increase with annual variations of 1.78 and 0.51%, respectively. Long-term exposure to these atmospheric pollutants poses a substantial threat to both the ecological environment and public health, with adverse outcomes reported across various disease categories, including those affecting the immune, respiratory, cardiovascular, and pulmonary systems, as well as hypertensive diseases in pregnancy and cancer (6–10). These adverse health impacts result in significant health losses and economic burdens (11–13). If left uncontrolled, air pollution-related fatalities are projected to escalate to 6–9 million globally by 2060 (14, 15). The link between air pollution and public health stems from human exposure to contaminated air. Therefore, a rigorous quantitative assessment of air pollution exposure is critical for evaluating associated health risks and serves as a fundamental basis for establishing ambient air quality standards and identifying priority areas for air pollution emission control. Air pollution exposure assessment is conducted at two levels: population and individual. Regional-scale population exposure evaluation relies on air pollutant concentrations and population distribution, while individual external exposure assessment involves tracking individual measurements or simulations. In both cases, air pollutant concentration plays a pivotal role in quantitatively assessing the extent of air pollutant exposure.

Air pollutant concentrations are conventionally monitored though automatic monitoring stations, offering objective and timely data on ambient air quality. However, the implementation of large-scale, grid-based, high-density air quality monitoring in cities often encounters challenges related to funding and deployment conditions. Addressing the influence of uneven distribution of regional pollution sources and variations in pollutant transmission conditions on urban areas, some studies have utilized spatial interpolation and land use regression techniques to simulate regional air pollution concentrations. However, it is important to knowledge that these methods are constrained by the limited number of monitoring stations and cannot provide comprehensive coverage of the entire population in the region. In recent years, satellite data has emerged as a valuable tool for monitoring air pollution concentrations and evaluating population exposure, boasting the advantages of wide spatial coverage and long-time observation capabilities (16, 17). Satellite remote sensing (SRS) offers a more effective approach to monitor and estimate atmospheric pollutant concentrations compared to ground-based monitoring methods. It provides valuable insights into the variation of pollutants across extensive weather maps, allowing for estimates of atmospheric parameters with broad spatial coverage. The rapid advancement of SRS technology, utilizing polar-orbiting and geostationary satellites, has significantly improved the spatial and temporal resolution of atmospheric environment monitoring. Beyond atmospheric environment monitoring, SRS technology finds extensive applications in monitoring various urban environments, such as the coverage of greenness, changes in land use, urban heat islands and so on. The mainstay of existing remote sensing (RS) health research lies in observing the relationship between the Normalized Difference Vegetation Index (NDVI) and human health, using NDVI as the primary variable for green spatial exposure (18). Multiple review analyses have demonstrated a positive correlation between greenness and physical activity and mental health (19, 20). Greenness prevents adverse mental health outcomes, cardiovascular disease, and mortality, and exposure to greenness may decrease depression levels and depressive symptoms (21, 22). However, conclusions drawn from a single influencing factor are not sufficient to account for all health conclusions, and greenness, as a mediator and effect modifier of associations with health, should be analyzed in combination with multiple influencing factors to draw more persuasive research conclusions.

This paper adopts a systematic review approach to elucidate technical principles of atmospheric SRSM and depict its progress in the field of atmospheric environmental epidemiology and health geography. The specific objectives of this study are as follows: (1) to visually analyze the literature using CiteSpace, thereby highlighting the progress of SRSM technology in environmental health-related research and identifying its prospects and limitations in the fields of atmospheric environmental health and geographical health research; (2) to conduct a comparative analysis of the advantages and limitations of SRSM technology and other monitoring methods in targeted fields; and (3) to explore the application of SRS technology in geographical health and the unique advantages it offers.

## Methods

### Literature searches

We conducted an extensive search of publications spanning from 1988 to the present in the CNKI, PubMed, Microsoft Bing, and Web of Science electronic databases. The search strategy encompassed a wide range of terms related to atmospheric pollutants (e.g., PM, PM_2.5_, ozone, nitrogen dioxide, sulfur dioxide, carbon monoxide, carbon dioxide, aerosols), urban air pollution (e.g., urban aerosols, pollution hotspots, indoor and outdoor air quality, air quality index), monitoring and modeling (e.g., air quality monitoring, air quality modelling, air pollutant concentration prediction), RS techniques (e.g., environmental RS, satellite monitoring, satellite imagery, satellite air pollution monitoring sensors, MODIS, multi-angle RS, atmospheric correction, multispectral imagery, high spatial resolution, polarimetric RS, dark target algorithms, deep blue algorithms, surface reflectance), data analysis and mapping (e.g., haze removal, meteorology, aerosol optical depth (AOD), aerosol modelling, GIS, pollution scenarios, spatial mapping, spatial analysis, spatial variability, spatial heterogeneity, spatiotemporal correlation, spatiotemporal patterns, multi-scale prediction), urban environment and health (e.g., urban health, health adaptation, urban scale, urban planning, urban morphology, built environment, population density, urban agglomerations, transport infrastructure, land use), machine learning techniques (e.g., machine learning, land use regression models, geo-weighted regression models, spatial regression models), and health outcomes (e.g., health symptoms, mental health, infectious diseases, health risk, health risk exposure assessment, infection rates, mortality rates, long-term trends). The main keywords are: urban air pollutants, environmental monitoring, SRS, satellite monitoring steps, urban environmental health, built environment, regression modeling, health risk. Additionally, we manually searched the references cited in each included article to supplement our initial search.

### Selection criteria

The inclusion criteria comprised studies (1) addressing the technical rationale and applications of SRS technology in atmospheric pollution monitoring; (2) focusing on geographical health research related to atmospheric pollution, influencing factors and approaches, and (3) exploring the substitutability of SRS technology in geographical health research methods. Exclusion criteria included research that (1) did not focus on SRS techniques and (2) did not concentrate on geographic health. After an initial screening based on titles and abstracts, 237 studies were selected. Following a review of the abstracts, 210 studies were further considered, and ultimately, after a thorough evaluation of the full texts, a total of 165 articles met all the criteria within the scope of this review.

### Analysis tools

For data collection, processing, and visualization, we employed CiteSpace, a widely recognized and popular data analysis software. CiteSpace facilitates a comprehensive understanding of historical and current information within a discipline, allowing efficient exploration and identification of research frontiers and emerging trends. Its essential functions streamline data processing and enhance accuracy. Researchers can utilize various analysis methods, such as word frequency analysis, mediated centrality analysis, co-occurrence analysis, cluster analysis, timeline mapping analysis, and emergent word analysis, all of which contribute to improved readability of the literature and identification of research frontiers (23). By identifying emerging keywords, this study aims to comprehend the progression of research hotspots and trends at specific time, detecting the rise or decline of particular subject terms or keywords. CiteSpace’s robust features and advantages have made it extensively utilized across diverse disciplines, such as online learning (24), hospitality research (24), and cross-cultural competence (25). Therefore, this study leverages CiteSpace for the present literature review.

## Results

### Visual analysis of the literature

**Figure 1:**
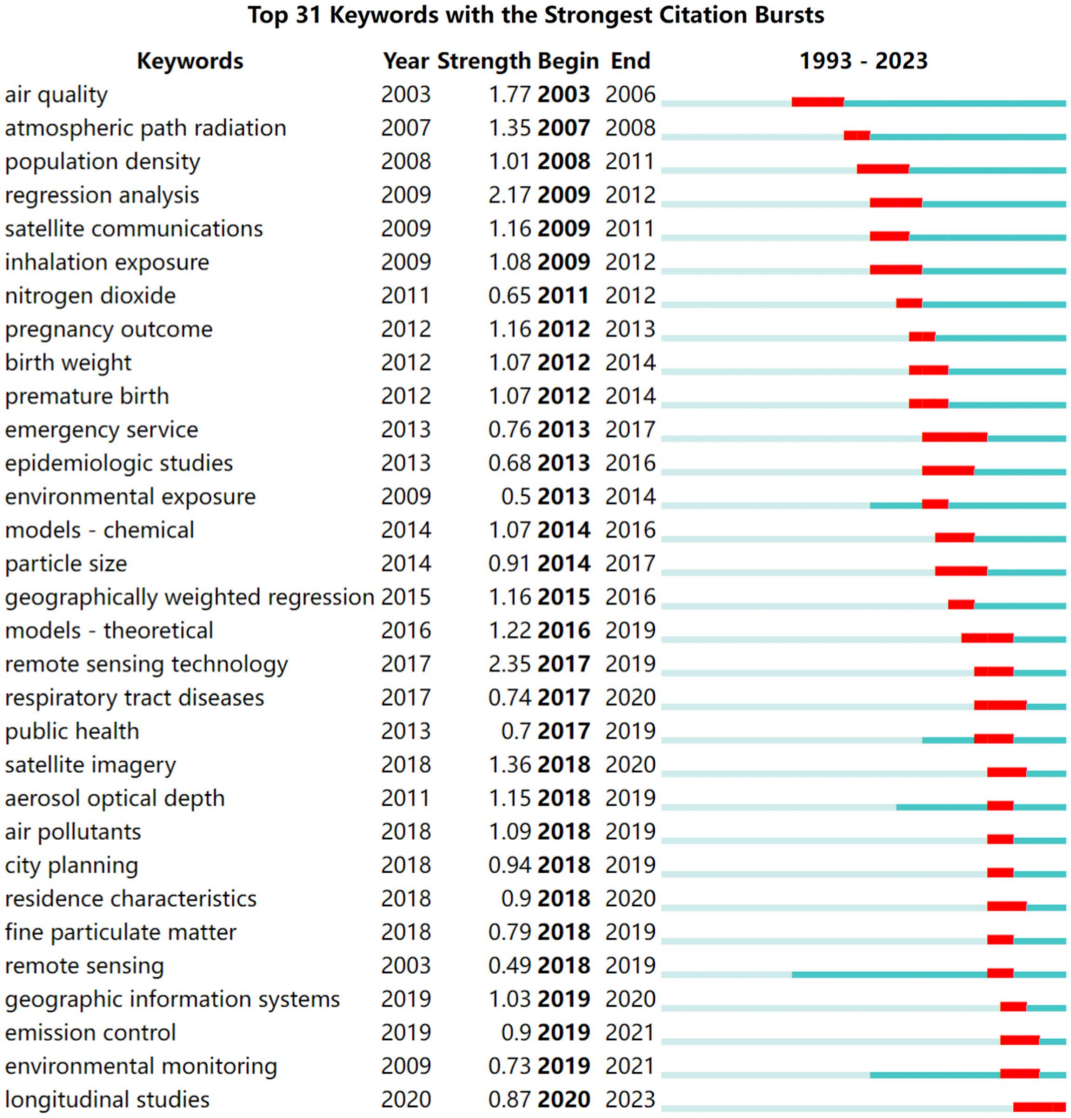
The emergent intensity and period of occurrence for the top 31 keywords.

**Figure 2:**
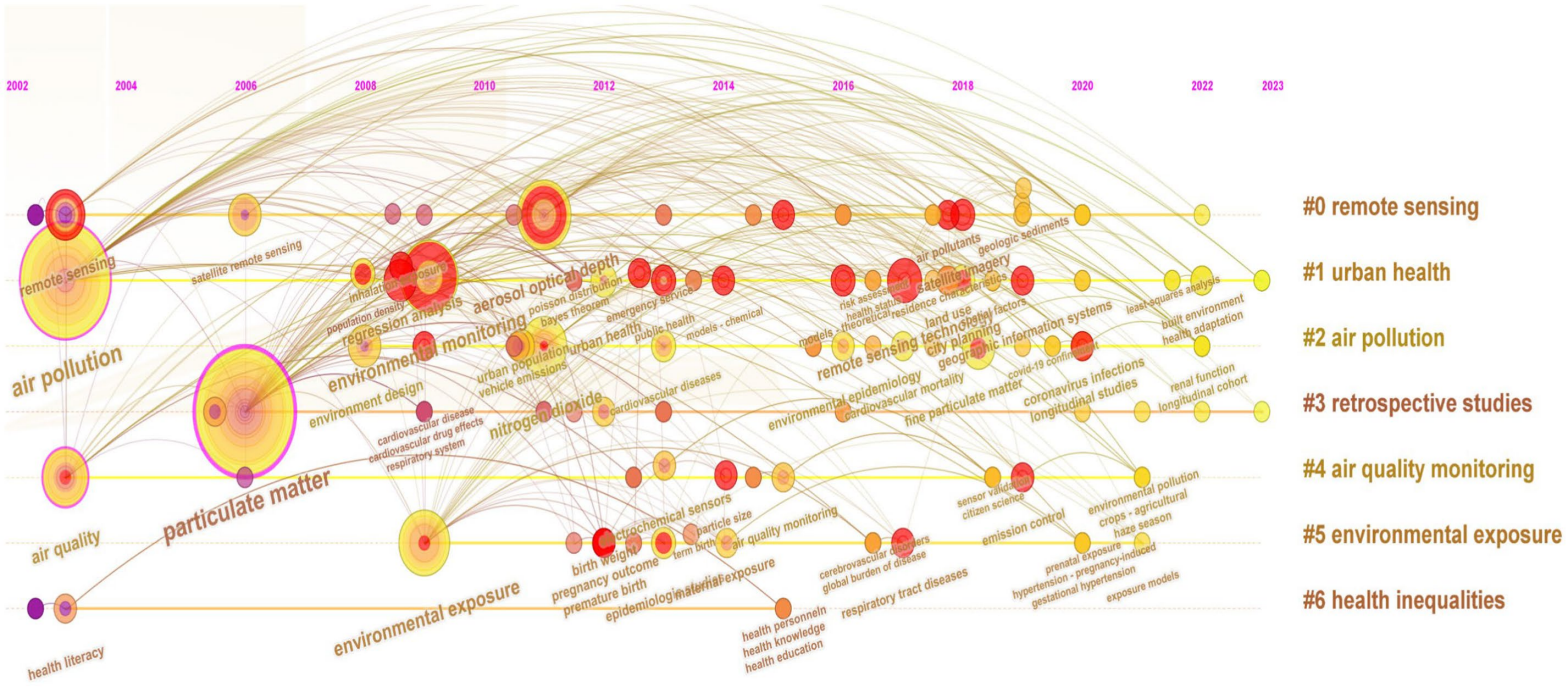
Timeline of keywords in the literature.

The literature search yielded a total of 190 articles encompassing the themes of SRS, built environment, and public health, spanning from 1988 to 2023. Employing the CiteSpace tool, we generated a temporal linear plot depicting the connections between keywords in the literature, allowing us to analyze the central themes and primary focuses of the relevant studies (26). Notably, the keywords “atmospheric RS” and “geographical health” co-occurred throughout the timeline. Table 1 and Figure 1 present the top 31 key terms with the highest hotspot, centrality, and burst intensity in the domains of RS and geographical health. To highlight their distinctive research attributes, we employed the Log-likelihood ratio (LLR) algorithm to obtain research terms and utilized the LLR algorithm’s labels for cluster identification. Furthermore, Figure 2 illustrated the 31 hot keyword nodes in red, showcasing their interconnections and relevance. According to Table 1, the keyword “particulate matter” emerged as the most frequent, appearing 40 times with a centrality of 0.32. Oher keywords like “air pollution” “environmental monitoring” “environmental exposure” “aerosol thickness” “air quality” “nitrogen dioxide” “RS techniques” and “SRS” displayed decreasing frequencies ranging from 38 to 4. Notably, keywords such as “particulate matter” “air pollution” “RS technology” and “environmental exposure” exhibited higher centrality, signifying their greater importance in terms of connectivity within the visualization network.

**Table 1:** The top 10 keywords ranked by frequency and centrality, respectively.

No	Frequency	Keywords	Centrality	Keywords
1	40	Particulate matter	0.32	Particulate matter
2	38	Air pollution	0.18	Air pollution
3	17	Environmental monitoring	0.15	Air quality
4	13	Environmental exposure	0.08	Aerosol optical depth
5	11	Aerosol optical depth	0.07	Electrochemical sensors
6	9	Air quality	0.05	Remote sensing
7	9	Nitrogen dioxide	0.04	Environmental monitoring
8	6	Remote sensing	0.03	Environmental exposure
9	5	Remote sensing technology	0.03	Nitrogen dioxide
10	4	Satellite remote sensing	0.03	Air pollutant

Figure 1 illustrates the prominent research frontiers in SRS technology that bridge the domains of atmospheric and built environment health, encompassing key themes such as “air pollution” “environmental exposure” “RS techniques” and “regression analysis and modeling.” These frontiers can be classified into four distinct periods based on the intensity of keyword bursts and the respective starting and ending years. Firstly, research focusing on air pollution and air quality has maintained its prominence since 2003. Studies initially emphasized PM and NO_2_ but gradually shifted toward investigating gaseous pollutants over the years. Secondly, within the realm of air pollution, researchers have directed their attention toward conducting epidemiological assessment of human exposure to polluted environments. Notably, an emerging area research at the forefront involves examining the interconnected health effects of factors such as population density, traffic, and land use in the context of human respiratory exposure. It is evident that the integration of SRS technology into studies of environmental exposure began to emerge around 2009. From 2017 onwards, SRS technology has experienced a surge in applications within the public health field, particularly concerning environmental concerns. This period has witnessed continuous technological advancements and adaptations, enhancing the utility of SRS for public health research. Lastly, the integration of diverse regression methods and models holds significant potential for enhancing the accuracy of pollutant data within a region, thus positively affecting the analysis and prediction of public health outcomes.

As evident from the more recent research frontier depicted in Figure 1 for the period of 2019–2021, notable terms such as “urban planning” “settlement characterization” and “environmental monitoring and RS technology” have emerged as prominent focal points. This shift signifies the gradual adoption of SRS technology in the field of geographical health, an emerging domain in recent years. SRS technology not only enables the capture of atmospheric pollutant composition but also effectively distinguishes between different land use types, street scenes, and categories within the built environment. The integration of atmospheric pollution and built environment health through SRS represents a major trend in the field of geographic and environmental health research, promising exciting avenues for future exploration.

Figure 2 outlines the evolution of keywords in included literature. A substantial corpus of research conducted between 2002 and 2004 centers on intricate air pollution monitoring methodologies, cutting-edge RS techniques, and the concomitant health perils entwined with air pollution. This epoch marked the acknowledgment of air pollution’s determinant role in urban public health. Initial air pollutant monitoring relied primarily on sparsely situated fixed monitoring stations worldwide (27). Primary methodologies for environmental exposure assessment encompassed conventional ground-based techniques and individual monitoring, leveraging real-time data emanating from stationary monitoring stations in diverse regions. This served as an indispensable approach for initial exposure analysis. However, the rapid pace of economic development catalyzed the emergence of independent individual monitors, yielding high-resolution, near-real-time data. These insights illuminated spatial air pollutant variations, enabling enhanced, personalized air quality monitoring. A pivotal moment in 2004 saw heightened attention on PM coinciding with the deployment of SRS for PM surveillance. Research between 2008 and 2010 extended to urban health’s environmental exposure, especially investigating inhalation exposure to PM across varying population densities. However, only limited studies delved into exploring the impact of PM on cardiovascular disease during this period. From 2010 to 2016, the synergy between AOD and PM emerged for expansive PM concentration extraction via SRS. Additionally, research on air pollutants expanded to encompass NO_2_ during this period. The rising concern surrounding exposure to ambient urban health and public health have gained considerable attention over the years due to the rising concern surrounding ambient air pollution led to an increase focus on urban health, with studies investigating not only respiratory exposure to PM but also particle diameter size and NOx in relation to cardiovascular disease, adverse pregnancy and birth outcomes, and various aspects of epidemiology. This trend contributed to the dissemination of health literacy and health education. Advanced SRS technologies in 2012–2016 revolutionized air pollution monitoring, allowing comprehensive urban health applications. Cardiovascular disease’s link to ambient air pollution retained its research significance. Combined RS with Geographic Information Systems technology, geographical factors like “settlement characteristics,” “green space use,” and “population density” emerged as influential on health. Post-2020, there has been a progressive refinement in the study and consideration of variables influencing urban health. Researchers have increasingly emphasized the incorporation of additional built environment variables, such as street design, land use, housing floor levels, and so on. Smaller-scale geographical health studies, including neighborhoods and industrial parks, have gained traction within the field of urban health. Mental health of urban populations has emerged as a focal point in the literature, signifying a growing recognition that urban health encompasses not only physical well-being but also mental health.

Bibliometric analysis reveals a growing focus on urban health issues related to air pollution since 2016. Early monitoring methods were limited by insufficient spatial and temporal coverage of ambient air quality information, often providing only supplementary data for polluted areas, resulting in limited representative area coverage. However, the incorporation of SRS technologies has enabled the inversion of pollution data at large spatial scales as well as extract images depicting changes in selected physical variables in cities over time. This advancement has strengthened the connection between air pollution and public health in the built environment in specific areas.

### Satellite remote sensing monitoring

SRSM has emerged as a promising solution to address data gaps in atmospheric monitoring. These low-cost air quality monitors with portability offer two types: one pertains to the sensor itself, and the other encompasses the complete monitoring system, including additional supporting hardware and software (28). These monitors contain multiple pollutant sensors within a single system (29). SRS technology, implemented with sensors on satellites, enables wide-scale air quality monitoring without direct physical contact with the atmosphere, overcoming limitations in regions where ground-based sensors are inaccessible. Researchers have effectively utilized satellite observation techniques to enhance or substitute the air pollution monitoring data obtained from existing stations. For instance, Yu et al. (30) utilized the Aura OMAERUV satellite aerosol product data (0.125 × 0.125°) to estimate ground-level air pollutant concentrations, demonstrating the reliability of satellite observations. Gupta et al. (31) found that satellite-derived AOD serves as a suitable proxy for monitoring PM_2.5_ air quality. Ju et al. (32) assessed atmospheric pollutants (O_3_, NO_2_, SO_2_, and HCHO) in Lanzhou. China, using Ozone Monitoring Instrument (OMI), revealing multidimensional characteristics and spatial distribution. Soleimany et al. (33) successfully mapped air pollutants’ spatial distribution by integrating various monitoring data encompassing Terra OMI (1 × 1°), Sentinel-5P (7 × 3.5 km^2^) and monitoring station data.

SRS technology offers real-time monitoring and forecasting of air pollution, along with mapping changes in air pollution. However, it is susceptible to tropospheric cloud interference, leading to incomplete data retrieval (34). To address these limitations, researchers are exploring numerical spatial interpolation/extrapolation techniques, such as inverse distance weighted, nearest interpolation, interpolating splines, and kriging (35, 36), which can estimate observations for non-monitoring points using known data from monitored points within the region (37). Chen et al. (38) employed developed a mixed-effects model and inverse distance weighted technique to replace missing AOD data, significantly reducing the missing rate from 87.9 to 13.8%. Ma et al. (39) introduced a novel geo Long Short-Term Memory (Geo-LSTM) model for generating spatial distributions of air pollutant concentrations, outperforming traditional methods.

SRSM excels in spatial scale and temporal coverage compared to ground monitoring and individual monitoring. Integrating ground observatory data, real-time air quality information, and various environmental variables, SRS offers a holistic view of the atmospheric conditions. Rigorous spatial interpolation and machine learning models are used for data validation and prediction of air pollution concentrations and air quality index (40). Despite challenges, ongoing improvements in SRS data help identify and mitigate elevated exposures in global communities. Moreover, high-resolution SRSM data has the potential to better address the global burden of disease resulting from environmental disparities compared to ground-based monitors. Table 2 shows a comparison of monitoring methods for atmospheric conditions.

**Table 2:** Comparison of monitoring methods for atmospheric conditions.

Monitoring methods	Principle	Distribution	Advantage	Disadvantage
Monitoring stations	High-Sensitivity Spectroscopic Techniques, Fourier Transform IR Spectroscopy, Fluorescence Spectroscopy Technique, Differential Optical Absorption Spectroscopy, Non-Dispersive Infrared Technology, Photoacoustic Spectrometer Technology (41, 42)	Large Urban and Small Peri-Urban Areas	24-Hour Continuous Online Monitoring and Construction of Heavily Polluted Areas	High Construction and Maintenance costs, Land Use Constraints, Sparse Network, Limited Regional Coverage
Individual monitoring	Scattered Light Principle and Changes in Specific Properties of Sensing Materials in the Presence of Gaseous Species	Randomness	Low-Cost, Portable, Real-Time Monitoring, Targeting Specific Population Groups for Monitoring Placement in Any Region	Short Monitoring Range, Low Sensitivity, Susceptibility to Missing Data, Challenges in Large-Scale Implementation
SRSM	Differential Optical Absorption Spectroscopy; Deep Blue Algorithms, Dark Target Algorithms, Structural Function, Polarization, Ultraviolet, Multiangle Algorithms et.al	Globally	Broad Coverage, Continuous Space-Based Observation, Enabling Real-Time Monitoring, Mapping of Pollution across the Earth’s surface, Extended Lifetime without physical contact with atmospheric pollutants	Meteorological Factors, Encounters Non-Random Missing Regional Data and Exhibits Discontinuities.

### Technical advancement of SRS for atmospheric environment monitoring

Technical advancements in SRS have revolutionized atmospheric environment monitoring, particularly in the field of aerosol substances. Aerosol SRSM began in the 1970s with the Multispectral Scanner (MSS) on ERTS-1 (Earth Resource Technology Satellite), enabling ground-based aerosol observations through the linear relationship between upwelling radiance and AOD (43). Over the years, various polar-orbiting satellite sensors, such as the AVHRR (Advanced Very High Resolution Radiometer), TOMS (Total Ozone Mapping Spectrometer), ATSR (Along Track Scanning Radiometer) sensors (including AATSR), the MODIS (MODerate-resolution Imaging Spectroradiometer) sensor on Terra (44), the MISR (Multi-angle Imaging Spectro Radio meter), POLDER (Polarization and Directionality of the Earth’s Reflectances), SeaWiFS (Sea-viewing Wide Field-of-view Sensor), MERIS (Medium Resolution Imaging Spectrometer), and the multi-angle polarimetric imager DPC (Directional Polarimetric Camera), China’s Gaofen-5 (GF-5) satellite (45), have been deployed to monitor aerosol products and atmospheric conditions. Notable studies have used SRS to assess air quality monitoring, track aerosol pollutant migration, and estimate ground-level air pollutant concentrations. For instance, the Global Aerosol Climate Project by NASA employed AVHRR satellite sensors in 1998 to achieve gridded monitoring of marine aerosols at a resolution of 1 × 1 (46, 47). In 2003, Hutchison utilized MODIS data from RS satellites with spatial resolutions of 250, 500, and 1,000 meters, as well as 10 kilometers, to assess air quality monitoring and forecasting in Texas and track the transport and migration of aerosol pollutants (48); Additionally, Wang et al. (49) retrieved global optical depth data for terrestrial aerosols from October to December 2018 using the DPC (3.3 km × 3.3 km) sensor onboard China’s GF-5, covering eight spectral channels ranging from 443 to 910 nm.

Continuous technological advancements have improved the spatial and temporal resolution of aerosol products. However, disparities in inversion algorithms and sensor sources may introduce variations in monitoring outcome (50–52). Polar-orbiting satellites equipped with atmospheric aerosol and PM monitoring sensors have been pivotal in achieving atmospheric environmental monitoring. The deployment of specialized sensors like MODIS, MISR, POLDER, OCTS, and OMI has significantly enhanced spatial resolution and allowed for simultaneous monitoring of both marine and terrestrial aerosols (53–60). SRS has gained international prominence as a widespread approach for monitoring atmospheric pollution. Computer-generated RS digital images of atmospheric radiance provide insights into atmospheric conditions, air quality, particle concentration, and hazardous gas presence (61–64). Aerosol inversion, an essential input parameter for atmospheric quality modeling and health-related studies, also aids in atmospheric correction of SRS images. The retrieval of AOD involves multiple steps, including radiometric and geometric corrections, and the influence of complex meteorological and anthropogenic factors on pollutant concentration (65). Table 3 summarizes commonly used aerosol monitoring sensors, including their respective satellites, spatial resolution, sensor characteristics, and applications.

**Table 3:** Satellite sensors for aerosol inversion (66–75).

Sensor	Satellite	Producing countries	Usage duration	Spatial resolution(km)	Attribute	Main application
AVHRR	NOAA-7, −9, −11, −14, −L, Metop-1	USA	1978–1994	1.1(local mode); 4.4(globe)	Long-term datasets	AOD
TOMS	Nimbus-7, Meteor-3, ADEOS, Earth Probe, QuikTOMS	USA	1978–1993;1991–1994	50	Long-term datasets; Sensitivity to absorbing aerosols on land and at sea	O_3_, SO_2_
POLDER	ADEOS, ADEOS II	France、Japan	1992–2002	7×6	Polarization is more sensitive to the refractive index of aerosols; Observation of Earth targets from 12 directions; Cloud screening using A-band, reflectivity threshold and spatial coherence	Aerosol properties measured by polarization
MISR	Terra	USA	2002 to present	17.6 × 17.6; 4.4 × 4.4	Flight calibration using high quantum efficiency diodes; Global coverage for 9 days	Aerosol
MODIS	Terra, EOS PM, Aqua	USA	1999 to present	0.25–1	High calibration accuracy; Large number of airborne band calibrators; Wide spectral range; Ability to detect clouds, shadows and heavy aerosols	H_2_O; cloud layers; Aerosol
OMI	EOS CHEM Aura	Finland, Netherlands Co-operation	2004 to present	13(local mode); 13 × 24(globe)	High calibration accuracy; Large number of on-board calibrators	O_3_; SO_2_; NO_2_; Aerosol; CHOCHO
AATSR/SLSTR	Envisat/Sentinel-3	European Space Agency(ESA)	2002–2012; 2016 to present	1	Dual-viewing angle (front view is 55°) Observation capability at different wavelengths; Can be used for atmospheric characterization and sea surface temperature	Aerosol; Land; Surface

### Main algorithms of atmospheric aerosol inversion by SRS

AOD that represents the transmittance rate through a vertical atmospheric column, severs as a quantitative indicator for atmospheric turbidity and total particle concentration (76). Nine algorithms, such as Dark Target(DT), Improved Dark Target, Structure Function, Multi-angle RS, Tandem method, Polarization RS, Deep Blue (DB), Cloud-top AOD, and Ground-air coupling, are employed for aerosol inversion, catering to variations in surface types and aerosol compositions (77–84). Supplementary Table S1 summarizes the detailed principles and applications of main algorithms. For instance, prior studies conducted by Jin et al. (85) and Chen et al. (86) utilized DT and improved DB algorithms, respectively, to estimate AOD in different regions, revealing broader coverage in hazy areas and higher AOD concentrations in urban areas and along highways. Moreover, Mukai et al. (87) conducted aerosol retrieval in hazy atmospheres using DT and polarization information from multiple viewpoints.

### SRS contribute to atmospheric particulate matter monitoring

**Figure 3:**
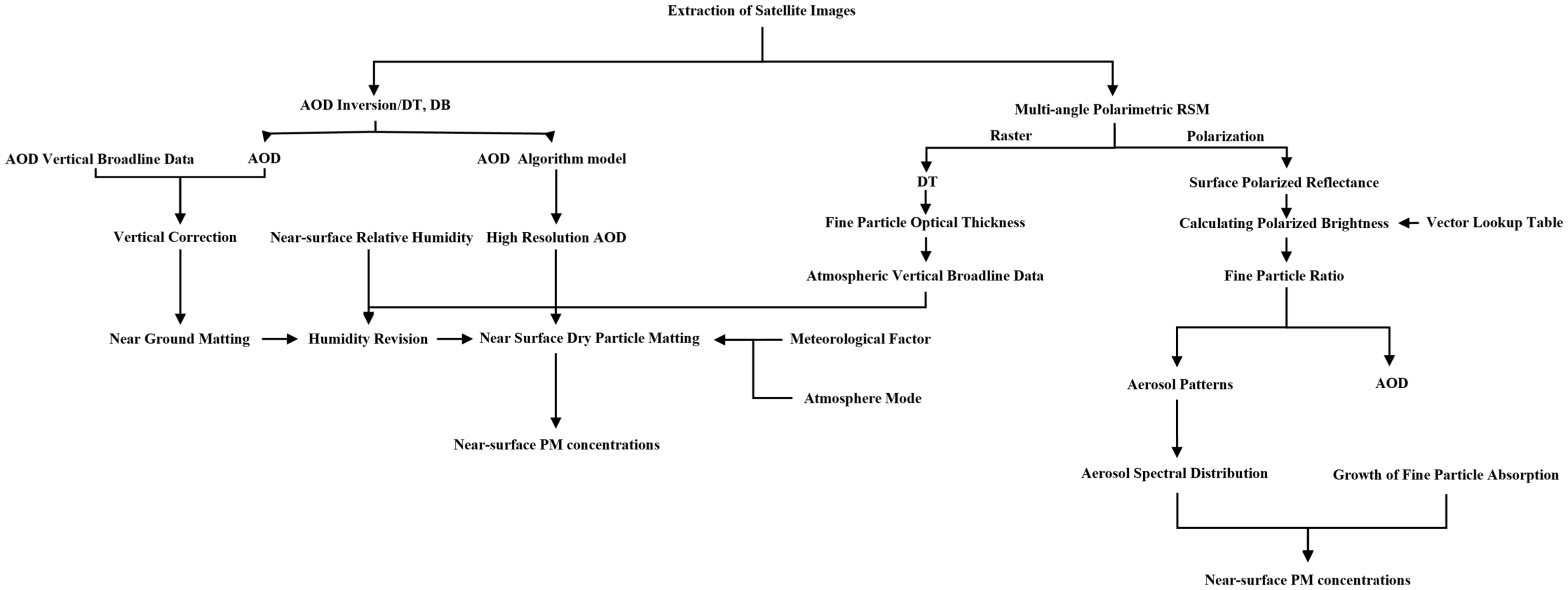
Inversion of AOD and transformation with PM.

SRS has significantly contributed to atmospheric particulate matter (PM) concentration inversion, particularly focusing on PM_10_ and PM_2.5_ measurements (Figure 3). Early studies relied on simple linear regression models to establish correlations between total column AOD and surface PM concentrations, assuming specific atmospheric conditions and aerosol properties (88–91). Assuming a dry and cloud-free sky, a mixed boundary layer with no overlapping aerosols at the boundary height (H), and aerosols with similar optical properties. The AOD can be expressed as follows equation (1) (88):


(1) $$AOD=\mathrm{PM}\times H\times f\left(RH\right)\times \left(\frac{{3Q}_{exr,\mathrm{dry}}}{4\rho {r_{ef}}_{f}}\right)$$

f(RH) denotes the ratio of the ambient extinction coefficient to the dry extinction coefficient; *Q_ext,dry_* represents the extinction efficiency under dry conditions; *ρ* indicates the mass density of the aerosol (g/m^3^); and *r_eff_* corresponds to the effective radius of the PM that calculated as the ratio of the third-order moment of the size distribution to the second-order moment.

However, researchers later extended these algorithms to more complex models, including land use regression, geographically weighted regression (GWR), nonlinear models, and machine learning approaches, to enhance inversion accuracy (92–96). For example, Hu et al. utilized a two-layer model (linear mixed model and GWR) to combine temporal and spatial variations, resulting in significantly improved PM_2.5_ distribution inversion accuracy in the southeastern United States (97, 98). Physicochemical models have also been proposed to calculate the relationship between AOD and PM_2.5_. For instance, Liu et al. introduced the scaling factor method, employing the Chemistry Transport Model (CTM) to simulate PM_2.5_ and AOD, and Zhang et al. developed a semi empirical formula based on the physical mechanism of mutual influence between aerosols, atmospheric PM, humidity, and temperature parameters (99–101). These models have effectively addressed the issue of uncertainty in PM_2.5_ concentration estimation, leading to improved precision in the results.

### Application of SRS in air pollutant monitoring

#### Application of SRS in monitoring atmospheric particulate matter

The analysis of spatial and temporal trends in atmospheric PM_2.5_ concentrations represents a significant application of SRS in atmospheric monitoring. For instance, a study in China harnessed satellite data from multiple satellites, including Aqua MODIS (10 × 10 km^2^), Terra MISR (17.6 × 17.6 km^2^) and OrbView-2 SeaWiFS (4 × 4 km^2^), along with ground-based PM_2.5_ measurements discern contamination patterns. The findings revealed an upward trend in China’s annual average PM_2.5_ concentration from 1998 to 2008, followed by a subsequent decrease (102). Similarly, in Korea, a comprehensive analysis leveraging various satellite sensors, namely Terra/Aqua MODIS (10 × 10 km^2^, Suomi-NPP VIIRS (Visible infrared Imaging Radiometer, 6 × 6 km^2^), Terra MISR (17.6 × 17.6 km^2^), and Aura OMI (13 × 24 km^2^)), in combination with ground-based observations, uncovered seasonal variations in PM_2.5_ concentrations using a multiple linear regression model (103). Furthermore, in the eastern United States, generalized linear models were employed, utilizing day-by-day PM_2.5_ concentration data from 2005 and MISR AOD (17.6 × 17.6 km^2^), to predict PM_2.5_ concentrations that demonstrated slight deviation from the observed values. The study also highlighted significant seasonal differences and spatial variability in MISR AOD values (104). Additionally, a collaborative investigation conducted in several countries (e.g., Australia, China, the Netherlands, etc.) integrated generalized additive modeling alongside a DT and DB inversion method. This approach was employed to retrieve Terra/Aqua MODIS (10 × 10 km^2^) AOD data, ground-based PM1 monitoring data, and other spatiotemporal predictors. The study indicated that the global PM1 concentration remained consistently high during the period of 2005–2014, establishing a robust association between PM1 exposure and health (105, 106). These comprehensive studies underscore the significance of integrating SRS inversion data with ground-based observations, thus providing a more holistic comprehension of long-term trends and high-resolution spatial variability of PM_2.5_ concentrations across diverse spatial and temporal scales. Moreover, the incorporation of influencing factors such as land use and meteorological conditions enhances the potential and accuracy of regional PM_2.5_ concentration predictions.

The continuous advancements in RS technology and global data assimilation systems have rendered SRS a valuable extension of ground-based networks, particularly in the realm of AOD measurements. The data sourced from satellites offer an independent and complementary data source to augment ground-based monitoring and computational modeling. This additional information serves as an extensive resource for ambient air quality monitoring, aiding in the determination of ground-level PM concentrations. Notably, it also contributes to refining the accuracy of population exposure data in public health atmospheric epidemiological studies, providing valuable insights into the impact of atmospheric conditions on human health. Therefore, SRS plays an indispensable role in complementing ground-based measurements, significantly enriching our understanding of atmospheric conditions and their implications for public health.

#### Application of SRS in monitoring atmospheric gaseous pollutant

SRS techniques have long been predominantly employed for monitoring atmospheric PM, however, they also hold promise for tracking other gaseous pollutants. Several noteworthy studies have demonstrated the efficacy of SRS in this context. In an American study, NOAA-10 and NOAA-12 satellites were used to monitor the spectral channel of brightness temperature (BT) difference between 11 and 8.3 μm (BT_8_-BT_11_), revealing the presence of sulfuric acid-water (H_2_SO_4_-H_2_O) aerosols in the stratosphere after the eruption of the Mount Pinatubo (107). Similarly, a German study utilized the differential optical absorption method with data to from the METOP-A series of satellites equipped with the Global Ozone Monitoring Experiment (GOME) sensors to analyze tropospheric NO levels. This investigation unveiled significant amounts of NO in the troposphere over Africa and in the outflow regions over the South Atlantic and Indian Oceans (108). Furthermore, a Chinese study leveraged the Tropospheric Monitoring Instrument (TROPOMI) board the Copernicus Sentinel-5 Precursor (S5P) satellite to examine O_3_ concentrations. By extracting NO_2_ concentrations and tropospheric HCHO column data, this study observed an acceleration in O_3_ production during the COVID-19 blockade in the North China Plain, particular in winter (109). Additionally, a Norwegian study combined data from Sentinel-5 TROPOMI and Terra/Aqua MODIS with on-site air quality monitoring data to extract NO_2_, O_3_, and PM_2.5_ concentration for 34 countries. The study revealed that due to the unprecedented reduction in global economic and transport activities during the COVID-19 blockade period, the concentrations of NO_2_ and AOD decreased, while that of O_3_ increased (110). While the current application of SRS for monitoring atmospheric gaseous pollutants may have some limitations, continuous advancements in SRS technology hold the potential for extracting more atmospheric pollutant data from satellite images in the future.

#### Application of SRS linking atmospheric environment to human health

SRS technology has emerged as a critical tool in environmental epidemiological studies, enabling researchers to investigate the impact of PM exposure on public health. In recent years, its application has expanded to encompass the monitoring of gaseous pollution, broadening its scope and relevance. In these studies, the monitoring of AOD and PM concentration data through satellite inversion has become an essential input parameter for assessing PM exposure among regional populations. By combing satellite AOD monitoring with representative parameters such as land use, researchers have constructed LUR and GWR models, facilitating regional population PM exposure evaluations. This approach has significantly improved the accuracy of SRSM in atmospheric environmental epidemiological studies. For instance, researches the United States and Canada utilized Terra and Aqua MODIS (10 × 10 km^2^) products to explore the association between PM exposure and population health. The findings revealed significant associations between AOD and PM10 and elevated daily events of cardiovascular disease and ischemic heart disease (111). Additionally, another study using AOD products and ecogeographic research methods explored the link between aerosol air pollution and chronic ischemic heart disease (CIHD). It observed a higher mortality rate from CIHD in the eastern United States, where AOD indicated higher levels of outdoor (112); Additionally, a study investigating the relationship between PM_2.5_ exposure and birth outcomes integrated satellite data and ground-based monitoring data generated by the PM_2.5_-AOD. It existed an association between PM_2.5_ exposure and adverse birth outcomes (113). The improved satellite accuracy has allowed for more comprehensive assessments. A Canadian study evaluated the contribution of long-term PM_2.5_ exposure to global mortality rates based on MODIS and MISR (0.1° × 0.1°) satellite monitoring data, which showed that 8.0% of adult deaths globally from cardiorespiratory disease, 12.8% from lung cancer, and 9.4% from ischemic heart disease were attributed to PM_2.5_ exposure (114); A US-Mexican study explored the relationship between MODIS (1 × 1km) measurements, ground station interpolated PM_2.5_ measurements, and acute respiratory symptoms (ARS) in children, identifying PM_2.5_ as a risk factor for the prevalence of ARS in children (115). In conclusion, SRSM for PM is one of the main data sources widely used in global atmospheric PM exposure assessment and environmental epidemiological studies.

In addition to the monitoring of atmospheric particulate pollutants, SRSM technology is gradually being applied to the monitoring of other gaseous pollutants with the development of gaseous pollution monitoring sensors. For instance, a study conducted in Hong Kong explored the correlation between long-term NO_2_ and O_3_ exposure and kidney health in Asian children and adolescents, basing on the retrieval of NO_2_ and O_3_ data from 2005 to 2018 in the TROPOMI secondary data. The findings revealed that the risk of chronic kidney disease increased by 7% for every 10 μg/m3 rise in the annual average NO_2_ concentration, while each 10 μg/m3 increase in the annual average O_3_ concentration resulted in a 19% reduction in the risk of chronic kidney disease (116); In another Chinese study, OMI (10 km × 10 km) inversion and monitoring data from the AURA satellite indicated that cardiovascular and respiratory deaths caused by O_3_ accounted for approximately 32 and 16% of all-cause mortality in 2016 (117); Similarly, a Swedish study utilized data from MetOp-A, MetOp-B, GOME-2 (50 × 50 km^2^), and OMI (13 × 24 km^2^) instruments on the Aura and Suomi satellites to extract SO_2_ vertical column densities for providing fast source estimates for dispersion modeling. The study conducted probabilistic analyses using a probit model and found that during the fires at the sulfur production site of Al-Mishraq, located 100 km from the center, there was a potential for respiratory symptoms, eye irritation, inflammation, burns, and fatalities at acute high concentrations any day (118). Based on the results of the appeal study, it was found that the potential health effects of gaseous pollutants at low resolution and small spatial scales may be underestimated. Utilizing satellite data can offer a clearer understanding of pollution data and spatial variations in public health at larger scales. As the accuracy of exposure levels directly affects the estimation of public health outcomes, the development of SRS data collection and modeling methods for high spatial and temporal resolution atmospheric pollutant exposure levels becomes a necessary and important future endeavor.

### Geographic health research

#### Frontier research linking public health with environment and air pollution

Urban layout, morphological distribution, and natural landscape distribution significantly influence population exposure to environmental air pollution and health behavior. In recent years, numerous studies have confirmed that the urban environment can impact the physical and mental health of urban residents by altering their exposure to environmental hazards and individual health behaviors. Most studies on the relationship between the built environment, air pollution and health have focused on extrapolating findings and predicting the health effects of air pollution on physical diseases. For instance, studies have associated low risks of non-communicable diseases, such as obesity, heart disease, and cancer, with favorable built environment exposures, such as a well-balanced land use mix, high residential densities, frequent walking behaviors, and extensive green spaces (119–124). Compact built environments are positively associated with infectious diseases (120). However, compact built environments may also generate positive exercise behaviors that reduce the risk of non-communicable diseases such as obesity, heart disease, and cancer (121–124). However, recent reports have begun investigating the relationship between urban form and mental health (125). Mental health is linked to urban surface design, often incorporating green measures (about 60%) or land use patterns (about 50%) (126). For example, high urban and residential densities may limit social interactions and lead to a sustained increase in negative psychological symptoms, such as anxiety, depression, and stress (127, 128). The quality of the living environment directly affects human health. Studies have demonstrated that the diversity of greenery, water areas, and land cover play crucial roles in reducing the impact of PM_2.5_, O_3_, and NO_2_ on mortality (129). Furthermore, the identification of five key urban form parameters (building density, mean building height, standard deviation of building height, mean building volume, and degree of enclosure) significantly influences the dispersion and distribution of pollutants in the neighborhood (130). However, determining parameters in these large domains often relies on modeling, which may lack timeliness and data authenticity. Modern research on the relationship between the built environment, air pollution, and public health requires large-area, high-precision photographic images, and pollutant concentration data. Therefore, SRS technology has rapidly developed in the field of geographic health research, providing timely images of the air pollutant spatial and temporal changes and satellite images of the built environment, and improving data completeness and accuracy at the same time. As a result, SRS technology plays a crucial role in enhancing the framework for studying the impact of urban air pollution exposure on population health. RSM briefly describes the results of land use, which greatly enriches the material for researching the effects of atmospheric environmental pollutants on population health.

#### Satellite technology utilized in geographical health

The most used RS data in environmental health studies are green space, such as the NDVI, and land use or land cover databases (131, 132). Green space exposure has been found to be closely related to the mental health of the public. A study in England revealed that pregnant women living in areas with higher NDVI were less likely to suffer from depression than those in areas with low green index (133). However, an investigation in Southern California, United States, utilized satellite measurements (Terra MOD13Q1, 250 m × 250 m), including NDVI, land-covered greenspace, and canopy cover, to assess the relationship between postpartum depression (PPD) and different types of green space exposure. The study found that the reduction in the risk of postpartum depression (PPD) was independent of NDVI and green space exposure (134). In addition to this, urban area characteristics in environmental health studies, such as the compactness of the built environment, unnatural infrastructure networks, modern transport systems, and air pollution conditions, lead to different exposure profiles in the population, making them potential risk factors for the spread and prevalence of various diseases across countries (135–137). However, in these complex situations, SRS can still be used to monitor urban air pollution. For example, a study in Indonesia collected NO_2_, SO_2_, and CO pollution data near communities through Sentinel-5P TROPOMI. It combined this data with socio-economic and health data from IFLS, land cover data from ESA CCI, and the GIS Spreading Index to measure the built environment. The study found that the quality of housing and neighborhood conditions played a role in the symptoms of lower respiratory infections (138). Similarly, Bechle (139) and colleagues examined the correlation between air pollution and built environment variables (population centrality, population density, transport availability, etc.), using OMI (11 × 11 km^2^) satellite estimates of NO_2_ concentrations and Landsat satellite imagery for defining the extent of the city. This approach suggests that changing urban form could be a potential strategy to mitigate urban air pollution and public health hazards.

In a New Zealand study, MODIS (medium resolution 500 m) surveillance was used to classify urban form, and tropospheric NO_2_ measurements from GOME, SCIAMACHY, and GOME-2 satellites. Three of the urban form metrics examined (proximity, roundness, and vegetation) showed a statistically significant relationship with urban NO_2_ and potentially had a large joint effect (140). Past literature has concluded that the association between the built environment and infectious diseases varies from region. Finer forms of control and data could be adapted to address the self-selection problem studied in each region. The incorporation of SRS discipline can provide more detailed spatial pollution and built environment imagery data that is regionally specific, which can be a valuable cross-fertilizer of different disciplines in the field of geographic health research. Supplementary Table S2 summarizes the satellites applied to geo-health research and provides a brief description of the satellite precision and the urban health areas in which they are applied.

## Discussion and conclusion

The concentration of atmospheric pollutant plays a pivotal role in air pollution exposure assessment. In particular om regional population exposure evaluation. Traditionally, air pollutant concentrations have heavily relied on on-site measurements from air pollution monitoring stations. However, these stations often tend to be situated in highly polluted areas, leading to spatial and temporal coverage issues and an imbalance in spatial distribution. Relying solely on ground-based monitoring data may introduce errors in calculating regional air pollutant concentrations, potentially affecting the accuracy of health impact studies related to air pollution. To address this challenge, researchers have incorporated SRS inversion techniques into existing methods to obtain regional monitoring data with large area coverage and high accuracy. Satellites offer advantages over ground-based monitoring, including spatial and temporal repetition, broad coverage, continuous observation in space, and global observation capabilities, which help to fill data gaps in ground-based monitoring and monitor air quality on a global scale in various countries and regions. The advancement of SRS sensors and retrieval algorithms has led to an increasing use of SRS products (including derivative AOD products) to monitor the impact of aerosols on the Earth-atmosphere system. However, the retrieval of satellite data is highly dependent on clear sky conditions, and cloud cover or cloudy day can obstruct data retrieval, resulting in incomplete datasets. Ground-level atmospheric pollutant concentrations are continuously monitored (e.g., hourly/daily/monthly/yearly), while AOD is retrieved only when satellites pass over a location (typically once a day). Consequently, measurements obtained at a specific location may not adequately represent diurnal variations at each site (141). However, Recent health impact studies have revealed the limitations of estimating relative risks solely based on ground-based information or satellite-derived exposure modelling (142). To address these limitations, studies now commonly combine data from both sources, correcting satellite data using ground-based monitoring data to achieve a high degree of precision, completeness, and broad coverage. Then, adding model simulations and predictive modelling are integrated into epidemiological exposure studies to prospectively assess health impacts, presenting a comprehensive approach in contemporary health research.

In recent years, SRS has proven successful in estimating atmospheric pollution concentrations in regions with limited ground-based monitoring data, by leveraging the correlation between satellite measurements and ground-based data (106, 143, 144). Various meteorological factors, including temperature, relative humidity, barometric pressure, wind speed, and sunlight variations due to cloud cover, influence the relationship between column aerosol loading measurements and near-surface dry PM_2.5_ mass concentrations (145, 146). Employing statistical modeling, satellite-retrieved AOD estimates historical and current PM exposure with good accuracy and spatial resolution by exploiting the relatively strong AOT- PM_2.5_ relationship (147). SRS technology has not only found utility in environmental monitoring but has also become an integral component of geo-health epidemiological studies related to exposure. There have been previous reviews showing that SRS technology can be applied in the field of environmental monitoring, in addition to its gradual incorporation into exposure-related geo-health epidemiologic studies. Earth observation (EO) satellites can capture a wide range of surface environmental factors on a large scale, providing fine-grained environmental measurements that complement the built environment factors commonly used in non-communicable disease (NCD) research (148, 149). However, the risk to human health is not limited to these built environment factors, and air pollution is the most critical component. Cardiovascular diseases, ischemic heart disease, chronic obstructive pulmonary disease, respiratory system issues, and adverse pregnancy outcomes are among the health concerns linked to outdoor air pollution (150, 151). Urban form and structure, urbanization and urban activities can also affect the generation and dispersion of air pollutants. Geographically informed individual-level exposure estimates are generated for geo-health epidemiological studies by incorporating land use maps, environmental pollutant concentrations (e.g., PM_2.5_, NO_x_, etc.) derived from aerial photographs and Landsat images, and other environmental and built environment factors (e.g., atmospheric conditions, topography, traffic flow, and population density). Therefore, the addition of atmospheric pollutant inversion techniques to the capture of various surface environmental factors with satellites may reveal previously overlooked risk factors that could better serve environmental public health research.

Prior to the development of RS technology, ground-based air pollution monitoring, classical maps, health reports, and databases of built-environmental variables used in health and geography studies and then combined air pollution, public health, and regionally based socio-demographic data, resulting in a limited breadth of findings. Instead, the impacts of built environment air quality and environmental exposures between neighboring cities can be explored by increasing the precision and scale of geospatial data to obtain highly credible research results. The rapid integration of SRS technology into geographic health research has resulted in timely acquisition of maps showing spatial and temporal variations of air pollutants, along with satellite images of the built environment. This advancement has significantly improved the completeness and accuracy of geographic data, allowing for more comprehensive studies on public health, considering the interplay between air pollutants and the built environment. Combining SRS with ground-based monitoring in epidemiological studies on the short/long-term health effects of ambient air pollutants provides a more detailed characterization of exposures. Additionally, incorporating models to predict outdoor air pollutant concentrations contribute valuable insights into the epidemiology, incidence, progression, and prediction of air pollutant-related diseases throughout individuals’ lifetimes (152). The cited literature through econometric calculations indicates that urban health issues related to air pollution have gained increasing attention since 2016, and the inclusion of SRS technology has strengthened the connection between air pollution and public health impacts of the built environment in specific regions. These multidisciplinary approaches serve as a valuable tool for assessing and predicting environmental health risks associated with air pollution. Moreover, it offers a platform to explore the potential benefits of SRS in geographic health research, leading to more precise findings in epidemiological studies. This integration also helps discovery potential future research directions in the field of air pollution epidemiology.

Based on the preceding discussion, this review advocates for increased utilization of diverse land use, meteorological, and physicochemical input models to provide small-grid or point-by-point predictions of key air pollutants at the local level, where SRS data are superior to *in situ* data for capturing human health impacts of air pollution. The selection of advanced AOD satellite data resolution and calibration algorithms should be prioritized to expand high-quality and fine-grained modeling to more regions, enabling a comprehensive understanding of various pollutant classes and major sources. By incorporating enhanced traffic data, new monitoring platforms, and machine learning techniques, pollutant prediction models have witnessed further improvements in performance and robustness for epidemiological studies. Furthermore, while most studies have analyzed the relationship between urban planning factors and air pollutant concentrations, it is feasible to combine highly accurate air pollution data from SRS and ground-based monitoring, as well as location-specific data from SRS, to investigate the effects of urban spatial structure, land use, spatial morphology, traffic, green space, and population density on long-term exposure to air pollutants (153, 154). In contrast, although many studies have investigated the relationship between physical characteristics of the built environment and environmental exposure, few studies have examined the type of surrounding built environment and building density based on environmental exposure at the transport site scale. Hence, by integrating air pollution data from satellite and ground-based monitoring, we can conduct research on the potential influence of traffic travel on the environmental exposure of nearby residents, the association between the built environment and travel patterns around the site, and the spatial relationship between environmental exposure from traffic travel and regional travel flow. Simultaneously, this approach opens a novel research avenue in geographic health to investigate the impact of multidimensional built environment characteristics and traffic travel flow. However, because sensors are mobile in space, the data they record for an area are usually discontinuous, leading to nonrandom missingness in contaminant retrieval results. The point in time and location at which a health exposure occurs is random in nature, and RS cannot guarantee complete monitoring of all exposure-related factors at the time of that exposure. At the same time, the actual indoor and outdoor exposure times cannot be precisely determined, and there are many occasions when indoor exposures are not monitored by RS. Diseases with long incubation periods have too many uncertainties (e.g., time of emergence, location of activity, etc.), which makes it even more difficult to assess and analyze them systematically using RS techniques.

In summary, atmospherically environmental epidemiology relies significantly on exposure assessment, often using atmospheric monitoring data directly as exposure proxies. However, the regional limitations of this method can lead to exposure prediction errors and misclassification of spatially heterogeneous pollutants. Studies have established a robust correlation between SRS estimates and ground-based measurements of key pollutants like PM_10_, PM_2.5_, and NO_2_. Yet, this necessitates extensive daily and regional calibration (155–157). Incorporating high-resolution (10 km-250 m) inverted pollution data from SRS with ground-based monitoring, air quality modeling and geographic predictors enhances health impact studies (158). Modern research at the nexus of the built environment, exposure to air pollutant, and health outcomes demands extensive, high-precision photographic imagery and pollutant concentration data. The rapid integration of SRSM into geo-health research holds promise for comprehending the pathways from built environment to health mediating by changes in exposure to atmospheric pollutants. Literature substantiated by econometric analyses highlights the effective integration of SRSM in concerning the health impacts of exposure to atmospheric pollutants derived from built environment alteration. This technology offers detailed spatial images of air pollution and built environments, enabling accurate temporal and spatial trend analysis of atmospheric air quality and geographic health research, fostering interdisciplinary collaboration. Nonetheless, SRSM data encounters inherent challenges, including discontinuous recording and incomplete retrieval under cloudy conditions. To address the limitations, numerical spatial interpolation/extrapolation can estimate non-monitoring observations through ground-based monitoring data. Thus, further research should enhance satellite performance and explore ground-sky integration (159). In essence, the synergy of SRSM and ground-based data holds significant promise for advancing our understanding of the links from atmospheric and built environment to human wellbeing’s. Further research should focus on refining satellite capabilities and optimizing combined data methodologies.

## Author contributions

YT: Writing – original draft, Writing – review & editing, Formal analysis, Visualization, Methodology. MD: Formal analysis, Data curation, Writing – review & editing. XC: Methodology, Conceptualization, Writing – review & editing. QZ: Funding acquisition, Writing – review & editing. ST: Writing – review & editing, Conceptualization. YL: Writing – review & editing, Data curation, Formal analysis. WW: Data curation, Writing – review & editing, Supervision.
