# Supplementary material for: Advancing application of satellite remote sensing technologies for linking atmospheric and built environment to health
Source: Front Public Health. 2023 Nov 15;11:1270033. doi: 10.3389/fpubh.2023.1270033 (PMC10690611; doi:10.3389/fpubh.2023.1270033)
Supplement: Supplementary file 1 [file Table_1.DOCX]

**Supplementary material**

**For**

**Advancing application of satellite remote sensing techniques for linking atmospheric and built environment to health**

Yuxuan Tian^1^, Mengshan Duan^1^, Xiangfen Cui^1*^, Qun Zhao^1^, Senlin Tian^1*^, Yichao Lin^2^, Weicen Wang^3^.

^1^ Faculty of Environmental Science and Engineering, Kunming University of Science and Technology, Kunming, Yunnan Province, 650500, China

^2^ Guizhou Research Institute of Coal Mine Design Co., Ltd., No. 48, Dazhi Road, Xibei Street, Huaxi District, Guiyang 550025, China

*^3^China Academy of Urban Planning Design, No. 5 Chegongzhuang West Road, Haidian District, 100044, Beijing*

*Corresponding author E-mails:

Xiangfen Cui: cui1987rainny@163.com,

Senlin Tian: tiansenlin@outlook.com

**Number of pages (including this page): 4**

**Number of Tables: 2**

**Table S1 summarizes the detailed principles and applications of main algorithms.**

| **Algorithm** | **Principle** | **Differences (characteristics)** | **Appearance date** | **satellites (environment)** |
| --- | --- | --- | --- | --- |
| Dark Target（DT） | Based on a linear relationship between surface reflectance in the blue, red, and infrared bands. | In areas with higher vegetation density, the surface reflectance is lower, and there is a stronger linear relationship between surface reflectance in the shortwave infrared, red, and blue bands in these regions. | Late 1990s by NASA | Landsant、MODIS、Sentinel |
| Improved Dark Target （IDT） | The IDT algorithm builds upon the concepts of the DT algorithm The key principle of the IDT algorithm involves exploiting the differences in surface reflectance between different satellite channels to estimate the aerosol signal. | Better handling of bright surfaces, Expanded spectral range, Considering the aerosol size distribution, Enhanced cloud screening, Higher spatial and temporal resolution | 2005 | Terra and Aqua MODIS |
| Structure Function | The principle behind using the Structure Function for AOD estimation involves analyzing the spatial variation and texture of an image to infer information about aerosol concentrations in the atmosphere. | Having a significant impact on the spatial structure of remote sensing images. Using to study phenomena in nature, such as the length distribution of rivers and the energy distribution of earthquakes. | 1969 | TM、AVHRR、SPOT、 |
| Multi-angle remote sensing | The principle behind multi-angle remote sensing is to exploit the variations in surface reflectance, scattering, and shadowing effects observed at different angles to derive valuable information about the properties of the target or scene. | Enhanced Accuracy, Aerosol Type Discrimination, AOD Variation Assessment, Cloud and Aerosol Separation, Correcting Surface Anisotropy, Atmospheric Correction Improvement, Urban Aerosol Studies | Late 1970s and early 1980s | Terra MISR、POLDER |
| Tandem method | It is based on the principle of combining measurements from two or more satellite sensors, each with different characteristics and wavelengths. | Advantage: Considering the differences in sensitivity to aerosols at various wavelengths to estimate AOD more accurately; Helping compensate for uncertainties and limitations in individual sensors.  Disadvantage: It requires a lot of computational resources and time. | 2002 | WorldView、SPOT、 |
| Polarization remote sensing | The principle behind polarization remote sensing lies in the fact that aerosols have the polarize sunlight as it passes through the atmosphere. When sunlight interacts with aerosol particles, it scatters in different directions and becomes partially polarized. | Advantage: This capability can enhance the accuracy of AOD retrieval and provide valuable information for understanding aerosol characteristics and their impacts on climate and air quality. It also can obtain the detail information on features.  Disadvantage: Requires a high level of technology and equipment, as well as a high level of data processing and analysis requirements. | 1999 | ALOS-2、PADARSAT、Terra SAR-X、Terra MISR |
| Deep Blue (DB) | The algorithm utilizes measurements from MODIS at different wavelengths, particularly in the blue and shorter-wavelength bands, where fine-mode aerosols have a more pronounced spectral dependence. These measurements are then combined with radiative transfer models to estimate the contribution of fine-mode aerosols to the total AOD observed by the satellite. | The key advantage of the Deep Blue algorithm is its ability to distinguish fine-mode aerosols from coarse-mode aerosols, which is particularly important in areas with bright surfaces where the contribution of fine-mode aerosols is significant. AOD can be inverted in desert, arid, semi-arid and urban areas. | 2005 | MODIS、SeaWiFS、VIIRS、GOCI |

**Table S2 summarizes the satellites applied to geo-health research.**

| **Satellite** | **Resolution** | **Applications in urban health** | **Examples** |
| --- | --- | --- | --- |
| Landsat | Spatial resolution of 30 m and a spectral resolution of 14 bits are achievable. | Green Space and Mental Health, , Land Use and Land Cover Changes, Air Quality Monitoring | Using Landsat-8 data to assess the association between green space exposure and mental health outcomes in urban populations. Green space during childhood is associated with better mental health (1). |
| Sentinel | Spatial resolutions in the range of 10 to 60 m and spectral resolutions between 12 to 16 bits are achievable. | Air Quality Monitoring, Vegetation and Green Spaces, Urban Expansion and Land Use Changes | Using Sentinel-2 data to assess the relationship between urban green spaces and nation’s happiness level in different countries (2). |
| MODIS | Spatial resolution ranging from 250 m to 1,000 m and 12 bits spectral resolution are achievable. | Air Quality and Health in Urban Areas, Vegetation and Green Spaces, Urban Expansion and Land Use Changes | Using MODIS AOD data to assess the association between fine particulate matter (PM2.5) exposure and cardiovascular disease in urban populations (Environmental Health Perspectives, 119(3), 367-372) (3). |
| GOES | Spatial resolution ranging from 1 km to 4 km and a spectral resolution of 10 bits are achievable. | Urban Heat Stress and Heat-related Illnesses, Air Quality Monitoring, Urban Green Spaces and Heat Mitigation, Urban Land Cover and Land Use Change, Extreme Weather Events and Health Impacts | Using MODIS (medium resolution 500m) surveillance in classify urban form, and tropospheric NO2 measurements from GOME, SCIAMACHY, and GOME-2 satellites. The urban form metrics examined showed a statistically significant relationship with urban NO2 and potentially had a large joint effect (4). |

Reference:

1. Engemann K, Pedersen CB, Arge L, Tsirogiannis C, Mortensen PB, Svenning JC. Residential green space in childhood is associated with lower risk of psychiatric disorders from adolescence into adulthood. *Proc Natl Acad Sci U S A*. (2019) 116(11):5188-5193. doi:10.1073/pnas.1807504116. Cited in: Pubmed; PMID 30804178.

2. Kwon OH, Hong I, Yang J, Wohn DY, Jung WS, Cha M. Urban green space and happiness in developed countries. *EPJ Data Sci*. (2021) 10(1):28. doi:10.1140/epjds/s13688-021-00278-7. Cited in: Pubmed; PMID 34094809.

3. Valdez RB, Al-Hamdan MZ, Tabatabai M, Hood DB, Im W, Wilus D, et al. Association of Cardiovascular Disease and Long-Term Exposure to Fine Particulate Matter (PM_2.5_) in the Southeastern United States. *Atmosphere.* (2021) 12(8). doi:10.3390/atmos12080947.

4. Bechle MJ, Millet DB, Marshall JD. Does Urban Form Affect Urban NO_2_ Satellite-Based Evidence for More than 1200 Cities. *Environ Sci Technol*. (2017) 51(21):12707-12716. doi:10.1021/acs.est.7b01194. Cited in: Pubmed; PMID 28898072.
